# Estratificação de Risco para Prevenção Primária de Morte Súbita Cardíaca em Cardiomiopatia Hipertrófica

**DOI:** 10.36660/abc.20201339

**Published:** 2021-07-15

**Authors:** Styliani Vakrou, Charalampos Vlachopoulos, Konstantinos A. Gatzoulis

**Affiliations:** First University Department of Cardiology and Electrophysiology Laboratory Hippokration” General Hospital; 1 National and Kapodistrian University of Athens School of Medicine Athens Grécia First University Department of Cardiology and Electrophysiology Laboratory, “Hippokration” General Hospital,National and Kapodistrian University of Athens School of Medicine,Athens - Grécia

**Keywords:** Cardiomiopatia Hipertrófica, Morte Súbita Cardíaca, Desfibriladores Implantáveis, Prevenção e Controle, Síncope

Lemos com grande interesse o artigo de Mattos et al. ^1^ sobre estratificação de risco na cardiomiopatia hipertrófica (CMH). ^1^ Os autores compararam as diretrizes de 2011 da American College of Cardiology Foundation/American Heart Association (ACCF/AHA) com as diretrizes de 2014 da European Society of Cardiology (ESC) em pacientes brasileiros com CMH e encontrou baixa concordância entre os dois sistemas. No total, 90 pacientes com CMH foram incluídos e 15 (17%) deles receberam um cardioversor desfibrilador implantável (CDI). Dois (2%) pacientes apresentaram choque apropriado, 6 (7%) morte súbita cardíaca (MSC) e 6 (7%) morte não cardíaca. De acordo com os critérios da ACCF/AHA de 2011, 43 (48%) pacientes receberam recomendação de Classe IIa para CDI, 3 (3%) receberam recomendação de Classe IIb e 44 (49%), Classe III. Se o escore de risco de MSC por MCH da ESC tivesse sido aplicado, 12 (14%) pacientes teriam recebido recomendação de Classe IIa para CDI (alto risco, ≥6%), 11 (12%) teriam recebido recomendação de Classe IIb (risco intermediário, ≥4% -<6%) e 67 (74%), Classe III (baixo risco, <4%). O coeficiente kappa calculado (0,355, p=0,0001) confirmou a baixa concordância entre as duas diretrizes. Especificamente falando, o modelo da ESC deixou desprotegidos todos os pacientes que apresentaram MSC ou abortaram MSC com apenas 2 deles (25%) sendo classificados como IIb. Os critérios da ACCF/AHA de 2011 recomendaram CDI (Classe IIa) para metade desses pacientes, com CDI sendo recomendado em muito mais pacientes do que os que realmente necessitavam. Os resultados deste estudo destacam a inadequação de estratégias precisas de estratificação de risco e contestam a tomada de decisão atual.

Esta não é a primeira vez que a precisão da predição de MSC na CMH provou ser insuficiente. ^2^ ^-^ ^4^ A CMH é a causa mais comum de MSC em jovens e a carga dessa perda devido à estratificação de risco não invasiva equivocada, por um lado, e a inserção desnecessária de um dispositivo, por outro, nos obrigam a melhorar esse quadro.

Nosso grupo propôs a contribuição adicional da estimulação ventricular programada (EVP) durante um estudo abrangente de eletrofisiologia (EFS) nas estratégias de estratificação de risco atuais, a fim de abordar seu baixo desempenho ^4^ . A população do estudo incluiu 203 pacientes com CMH, e como no estudo de Mattos et al., ^1^ a maioria foi avaliada como risco baixo a intermediário para MSC (60% apresentava um único fator de risco). Implantou-se CDI em 92 (45,3%) pacientes, com ocorrência de desfecho primário (MSC e/ou terapias apropriadas com CDI) em 20 pacientes (9,9%). O achado importante do estudo foi que a EVP foi indutível em todos, exceto um paciente que apresentou o desfecho primário, ao passo que as diretrizes da AACF/AHA de 2011 e da ESC de 2014 classificaram erroneamente 3 e 9 pacientes, respectivamente. Em nossa população, as diretrizes de 2011 da ACCF/AHA teriam levado à implantação de 171 CDIs, enquanto as diretrizes da ESC teriam levado à implantação de 53, e, somente a EVP, em 79 pacientes. A combinação de cada uma dessas diretrizes com o protocolo EVP aumentaria esses números para 187 e 110 pacientes, respectivamente, sem perder nenhum desfecho primário. Principalmente em conjunto com as diretrizes da ESC, a sensibilidade e a especificidade ideais foram alcançadas da maneira mais econômica. Outra vantagem importante da EFS acabou sendo a caracterização adequada do mecanismo da síncope.

O uso da EVP na estratificação de risco de prevenção primária para MSC na CMH é a Classe III nas diretrizes europeias de 2014 e nas diretrizes da ACCF/AHA de 2011, com o principal argumento sendo os riscos de tal procedimento invasivo e seu custo, com base no nível não suportado de evidência C. No entanto, nosso método foi viável e seguro em todos os casos, enquanto a classificação incorreta ou a implantação inadequada de CDIs são muito mais desoladoras e caras. O baixo desempenho das diretrizes da ESC e da ACCF/AHA pode ser devido a sua incapacidade de expressar o mecanismo exato de arritmogênese nesta doença. A ressonância magnética cardíaca (RMC) tem sido uma importante ferramenta na avaliação da CMH, uma vez que a presença de fibrose de realce tardio com gadolínio é considerada um forte preditor independente para arritmias ventriculares malignas. ^5^ O uso de achados da RMC na estratificação de risco foi considerado nas diretrizes de 2020 da ACC/AHA.

Em conclusão, a identificação adequada de pacientes com maior risco de morte súbita indicados para a terapia com CDI, que salva vidas, com controle de tratamento excessivo, continua sendo o Santo Graal da CMH. Mattos et al., ^1^ apresentam evidências adicionais na literatura atual sobre as inadequações dos escores de risco e destacam a necessidade de introdução de critérios mais sensíveis e específicos. A estimulação ventricular programada pode ser uma nova ferramenta salvadora ao nosso arsenal contra a morte cardíaca súbita.

## Carta-resposta

A morte súbita cardíaca (MSC) é considerada a complicação mais dramática da cardiomiopatia hipertrófica (CMH), com uma incidência anual estimada de 0,5–1%. ^1^ Ao longo dos anos, ACC/AHA e ESC publicaram diretrizes de consenso que apresentam diferentes abordagens para a estratificação de risco de MSC com base em preditores clínicos independentes. Apesar da acurácia relativamente baixa desses marcadores, as estratégias atuais teriam razoável poder discriminatório para o reconhecimento de pacientes de alto risco. No entanto, a avaliação prognóstica é um desafio na CMH, principalmente em pacientes de risco baixo a moderado. A MSC pode surgir na ausência de fatores de risco conhecidos e algumas abordagens mostram resultados conflitantes em diferentes populações. ^2^ Além disso, as discrepâncias metodológicas entre as diretrizes norte-americanas e europeias podem determinar níveis discordantes de recomendação em relação ao cardioversor desfibrilador implantável (CDI) na prevenção primária. ^3^ Portanto, a estratificação de risco ainda é imprecisa e requer maior investigação.

O artigo de 2018 de Gatzoulis et al., ^4^ reavalia o papel do estudo eletrofisiológico para a avaliação prognóstica em uma coorte unicêntrica de CMH de risco baixo a intermediário, estratificada de acordo com as diretrizes atuais. Os autores concluem que a estimulação ventricular programada (EVP) combinada com os modelos atuais acrescenta sensibilidade e valor preditivo negativo à estratificação de risco. ^4^ Os resultados são interessantes, mas precisam ser confirmados em estudos prospectivos maiores. A EVP foi reduzida à classe III nas diretrizes atuais devido à baixa sensibilidade e aos possíveis riscos. Da mesma forma, o fracionamento dos eletrogramas do ventrículo direito tem sido relacionado à MSC, mas não foi assimilado devido à natureza invasiva do procedimento, o que pode afetar sua realização em alguns casos. ^5^ A instabilidade elétrica é transitória na CMH e deve ser avaliada periodicamente assim como outros preditores com características dinâmicas. ^2^ ^,^ ^5^ Deste modo, as diretrizes recomendam a reavaliação do risco de MSC durante o seguimento. Avaliação clínica não invasiva e de fácil aquisição favorece a abordagem de rotina do paciente.

Na última diretriz da ACC/AHA de 2020, foi introduzida um algoritmo com três novos preditores não invasivos: fibrose na forma de realce tardio através de ressonância magnética cardíaca com gadolínio, aneurisma apical do ventrículo esquerdo e fração de ejeção reduzida. ^1^ Todos estes estão direta ou indiretamente relacionados a alterações na arquitetura miocárdica, principalmente a fibrose de substituição. Há uma tendência para a assimilação de novas e mais precisas tecnologias não invasivas para a avaliação prognóstica de rotina na CMH projetada para um futuro próximo. Talvez os protocolos invasivos entrem em desuso com base em avaliações de custo-efetividade. No entanto, a EVP pode ser aplicada em casos com estratificação de risco discordante entre as diretrizes em relação à recomendação de CDI, podendo contribuir em casos com síncope cardíaca inexplicada.

Por fim, a avaliação do desarranjo celular com tensor de difusão por ressonância magnética cardíaca, genotipagem, perfil de microRNAs e outros biomarcadores promissores, bem como a avaliação não invasiva de isquemia miocárdica, poderão assegurar uma avaliação mais rigorosa do substrato anatômico e elétrico da doença. ^2^ ^,^ ^6^ Porém, sua assimilação à prática clínica diária deve levar em consideração os recursos disponíveis localmente e representará um grande desafio. A busca por novos paradigmas na CMH está em curso e uma avaliação eletrofisiológica mais precisa dos mecanismos da MSC é necessária, podendo complementar ou mesmo suplantar a estratificação de risco de atual.

**Beatriz Piva e Mattos**

**Fernando Luís Scolari**

**Henrique Ianhke Garbin**
