# Supplementary material for: Estratificação de Risco para Prevenção Primária de Morte Súbita Cardíaca em Cardiomiopatia Hipertrófica
Source: Arq Bras Cardiol. 2021 Jul 15;117(1):157–9. [Article in Portuguese] doi: 10.36660/abc.20201339 (PMC8294717; doi:10.36660/abc.20201339)
Supplement: Translation

# Translation: Risk Stratification for Primary Prevention of Sudden Cardiac Death in Hypertrophic Cardiomyopathy

**Authors:** Styliani Vakrou, Charalampos Vlachopoulos, Konstantinos A. Gatzoulis

**Affiliations:** 1 Greece First University Department of Cardiology and Electrophysiology Laboratory, “Hippokration” General Hospital, National and Kapodistrian University of Athens School of Medicine,Athens - Greece

**Keywords:** Hypertrophic Cardiomyopathy, Sudden Cardiac Death, Defibrillators, Implantable, Prevention and Control, Syncope

We have read with great interest the article by Mattos et al. ^1^ regarding risk stratification in hypertrophic cardiomyopathy (HCM). ^1^ The authors compared the 2011 American College of Cardiology Foundation/American Heart Association (ACCF/AHA) guidelines with the 2014 European Society of Cardiology (ESC) guidelines in Brazilian HCM patients and found low agreement between the two systems. A total of 90 patients with HCM were included and 15 (17%) of them received an implantable cardioverter-defibrillator (ICD). Two (2%) patients experienced appropriate shock, 6 (7%) experienced sudden cardiac death (SCD) and 6 (7%) had non-cardiac death. According to the 2011 ACCF/AHA criteria, 43 (48%) patients received Class IIa recommendation for ICD, 3 (3%) received Class IIb and 44 (49%), Class III. If the ESC HCM risk-SCD score was applied, 12 (14%) patients received Class IIa recommendation for ICD (high risk, ≥6%), 11 (12%) received Class IIb (intermediate risk, ≥4%-<6%) and 67 (74%), Class III (low risk, <4%). The calculated kappa coefficient (0.355, p=0.0001) confirmed the low agreement between the two guidelines. In specific, the ESC model left unprotected all patients who experienced SCD or aborted SCD with only 2 of them (25%) being classified as IIb. The 2011 ACCF/AHA criteria recommended ICD (Class IIa) for half of these patients, but an ICD was recommended in much more patients than actually needed. The findings of this study highlight the inadequacy of precise risk stratification strategies and challenge current decision making.

This is not the first time that the accuracy for prediction of SCD in HCM is proven to be underpowered. ^2^ ^-^ ^4^ HCM is the most common cause of SCD in young individuals and the burden of such a loss due to wrong non-invasive risk stratification on the one hand, and the unnecessary insertion of a device on the other, urge us to do better.

Our group proposed the additional contribution of programmed ventricular stimulation (PVS) during a comprehensive electrophysiology study (EPS) in the current risk stratification strategies in order to address their underperformance. ^4^ The study population included 203 HCM patients, and like in the study by Mattos et al., ^1^ the majority were low-to-intermediate risk for SCD (60% had a single risk factor). An ICD was implanted in 92 (45.3%) patients and the primary endpoint (SCD and/or appropriate ICD therapies) occurred in 20 patients (9.9%). The important finding of the study was that PVS was positive in all but one patient who experienced the primary endpoint, while the 2011 AACF/AHA and the 2014 ESC guidelines misclassified 3 and 9 patients, respectively. In our population, the 2011 ACCF/AHA guidelines would have led to the implantation of 171 ICDs, while the ESC guidelines would have led to the implantation of 53, and PVS alone in 79 patients. The combination of each of these guidelines with the PVS protocol would increase these numbers to 187 and 110 patients, respectively, without missing any primary endpoint. Especially when combined with the ESC guidelines, optimal sensitivity and specificity was achieved in the most cost-effective manner. Another important advantage of EPS turned out to be the proper characterization of syncope mechanism.

The use of PVS in risk stratification of primary prevention for SCD in HCM is Class III in the 2014 European and the 2011 ACCF/AHA guidelines, with the main argument being the risks of such an invasive procedure and its cost, based on unsupported level of evidence C. However, our method was feasible and safe in all cases, while misclassification or inappropriate implantation of ICDs is much more devastating and costly. The underperformance of the ESC and ACCF/AHA guidelines may be due to their inability to express the exact mechanism of arrhythmogenesis in this disease. Cardiac magnetic resonance imaging (CMR) has been a valuable tool in the evaluation of HCM, as the presence of late gadolinium enhancement fibrosis is considered a strong independent predictor for malignant ventricular arrhythmias. ^5^ The use of CMR findings in risk stratification has been taken into consideration in the 2020 ACC/AHA guidelines.

In conclusion, proper identification of patients at highest risk for sudden death deserving lifesaving ICD therapy, while controlling overtreatment, remains the holy grail in HCM. Mattos et al. ^1^ provide additional evidence in the current literature of the inadequacies of risk scores and highlight the need for the introduction of more sensitive and specific criteria. Programmed ventricular stimulation could be a life-saving addition to our armamentarium against sudden cardiac death.

## Reply

Sudden cardiac death (SCD) is considered the most dramatic complication of hypertrophic cardiomyopathy (HCM) with an estimated annual incidence of 0.5–1%. ^1^ Over the years, the ACC/AHA and the ESC have published consensus guidelines providing different approaches for SCD risk stratification based on independent clinical predictors. Despite the relatively low accuracy of these markers, current strategies are considered to have a reasonable discriminatory power for the recognition of high-risk patients. However, prognostic assessment is challenging in HCM, especially in low to moderate risk patients. SCD may arise in the absence of known risk factors and some approaches show conflicting results in different populations. ^2^ Moreover, methodological discrepancies between the North American and the European guidelines may determine discordant levels of recommendation regarding implantable cardioverter defibrillator (ICD) in primary prevention. ^3^ Therefore, risk stratification is still imprecise and requires further investigation.

The 2018 manuscript of Gatzoulis et al. ^4^ reevaluates the role of electrophysiological study for prognostic assessment in a low to intermediate risk single-center HCM cohort stratified according to contemporary guidelines. The authors conclude that programmed ventricular stimulation (PVS) combined with current models add sensitivity and negative predictive value to risk stratification. ^4^ Results are interesting but need to be confirmed in larger prospective trials. PVS has been downgraded to class III in current guidelines due to the low sensitivity and potential risks. Similarly, fractionation of right ventricle electrograms has been related to SCD but was not assimilated due to the invasive nature of the procedure, which may impact feasibility in some settings. ^5^ Electrical instability is transitory in HCM and should be periodically evaluated such as other predictors with dynamic characteristics. ^2^ ^,^ ^5^ The guidelines recommend the reassessment of SCD risk during follow-up. Clinical and more easily acquired non-invasive approaches favor routine patient evaluation.

In the recent 2020 ACC/AHA guideline, an algorithm was introduced with three novel non-invasive predictors: late gadolinium enhancement fibrosis on cardiac magnetic resonance, left ventricular apical aneurysm and reduced ejection fraction. ^1^ All of them are directly or indirectly related to the impaired myocardial architecture, mainly replacement fibrosis. There is a trend towards the assimilation of novel and more accurate non-invasive technologies for routine HCM prognostic evaluation projected for the near future. Perhaps, invasive protocols will be gradually neglected to the past based on a cost-effectiveness appraisal. However, PVS may find a role in cases with discordant risk stratification between guidelines regarding ICD recommendation, and may contribute in cases with cardiac-related unexplained syncope.

Finally, the cell disarray evaluation with diffusion tensor cardiac magnetic resonance imaging, genotyping, microRNA profile and other promising biomarkers, as well as the non-invasive assessment of myocardial ischemia, will assure a more reliable assessment of the anatomical and electrical substrate of the disease. ^2^ ^,^ ^6^ Nevertheless, their incorporation into daily clinical practice should take into account locally available resources and will certainly represent another great challenge. A search for new paradigms is in course in HCM and a more precise electrical evaluation of SCD mechanisms is needed and may add or even outperform our current risk-stratification.

**Beatriz Piva e Mattos**

**Fernando Luís Scolari**

**Henrique Ianhke Garbin**
